# Mothers’ and fathers’ media parenting practices associated with young children’s screen-time: a cross-sectional study

**DOI:** 10.1186/s40608-018-0214-4

**Published:** 2018-12-03

**Authors:** Lisa Tang, Gerarda Darlington, David W L Ma, Jess Haines, on behalf of the Guelph Family Health Study

**Affiliations:** 1 Department of Family Relations and Applied Nutrition, University of Guelph, Guelph, ON Canada; 2 Department of Mathematics and Statistics, University of Guelph, Guelph, ON Canada; 3 Department of Human Health and Nutritional Sciences, University of Guelph, Guelph, ON Canada

**Keywords:** Parenting practices, Childhood obesity, Screen time, Sedentary behaviour

## Abstract

**Background:**

A major contributor to the growing obesity crisis is screen based sedentary behaviour among young children. Media parenting practices may be an important determinant of children’s screen time, however published research exploring the influence of parenting practices on children’s screen time has mainly focused on children’s television use and the parenting practices of the mother. This study examined children’s use of mobile media devices (as well as television use) and included the role of fathers in media parenting, allowing a fuller understanding of the role mothers’ and fathers’ media parenting practices play on children’s screen time in the current media environment.

**Methods:**

Secondary data analysis was conducted using data from 62 children between 1.5 and 5 years of age and their parents (39 mothers, 25 fathers), who were part of the Guelph Family Health Study - phase 2 pilot. Linear regression using generalized estimating equations was used to examine associations between media parenting practices and children’s weekday and weekend screen-time.

**Results:**

Mothers’ screen-time modeling, mealtime screen use, and use of screens to control behaviour were positively associated with children’s weekday screen-time. Mothers’ practices of monitoring screen-time and limiting screen-time were inversely associated with children’s weekday screen-time. Fathers’ mealtime screen use was positively associated with children’s weekday screen-time; whereas fathers’ monitoring screen-time and limiting setting were inversely associated with children’s weekday screen-time. Fathers’ modeling and use of screens to control behaviour was not significantly associated with children’s weekday screen time. While most associations were similar for weekend day screen time there were a few differences: Fathers’ use of screens to control behaviour was positively associated with children’s weekend screen-time. Mothers’ and fathers’ modeling and mealtime screen use were not significantly associated with children’s weekend screen time.

**Conclusion:**

Mothers’ and fathers’ media parenting practices were associated with children’s screen-time. Interventions aimed at reducing children’s screen-time should address both mothers’ and fathers’ media parenting practices.

## Background

The World Health Organization (WHO) recognizes childhood obesity as a growing epidemic [1]. The prevalence of children with obesity and overweight under the age of five worldwide is estimated to be 41 million [1]. In Canada, the rate of children with obesity is on the rise with nearly one third of children being categorized as having overweight or obesity [2]. A major contributor to the growing obesity crisis is screen based sedentary behaviour among young children [3–5]. Both observational and intervention research has shown that higher levels of screen time, typically measured as TV viewing, is associated with increased risk of obesity among children [6, 7]. With only 15% of Canadian preschoolers meeting the Canadian Sedentary Behaviour Guidelines for the Early Years of less than 1 h of recreational screen time per day [8, 9], identifying effective strategies to reduce young children’s screen time is needed. To inform such strategies, we must first understand key determinants of children’s screen time.

Parents have been identified as playing a critical role in the development of their young children’s weight-related behaviours, including screen time [10]. In particular, media parenting practices, defined as the specific methods parents employ to guide the media use of their children, may be an important determinant of children’s screen time. Research related to media parenting practices to date has focused on children’s television time with few studies including mobile media devices such as tablets and smartphones [11, 12]. These mobile media devices have soared in popularity among young children in recent years; from 2011 to 2013 the percentage of 2 to 4-year-old children using mobile media devices in the United States increased from 39 to 80%, while television viewing time decreased [13]. Thus, research aiming to understand how media parenting practices are associated with children’s screen based sedentary behaviours in this current media environment must include screen-time assessments that take into consideration time spent on mobile media devices as well as more traditional modes of screen time.

There is also limited representation of fathers in the current research related to media parenting practices and children’s screen time. Aftosmes-Tobio et al. [11] examined 103 studies in their systematic review of media parenting and childhood obesity and found that only 57 of these studies included fathers. The majority of these studies that did include fathers combined information across parents rather than distinguish mothers from fathers. Thus, little is known about how fathers’ parenting practices influence children’s screen time. The underrepresentation of fathers is particularly concerning given emerging research identifying fathers as key stakeholders in childhood obesity prevention. A prospective study found that children with an obese father and a healthy weight mother were over 10 times more likely to be obese 4 years later than children with two healthy weight parents, whereas the same pattern was not observed for children with an obese mother and healthy weight father [14]. This finding underscores the need to understand fathers’ role in the development of childhood obesity-related behaviours, including children’s screen time.

Using data from the Guelph Family Health Study (GFHS), a family-based cohort study in Ontario, Canada, this study examined the associations between both mothers’ and fathers’ parenting practices (screen time modeling, mealtime screen use, use of screens in the bedroom, limit setting, use of screens to control behaviour, and screen time monitoring) and weekend/weekday screen time (television, computers, videogames, tablets and smartphones) among young children aged 1.5 to 5 years.

## Methods

### Recruitment and eligibility

**Fig. 1:**
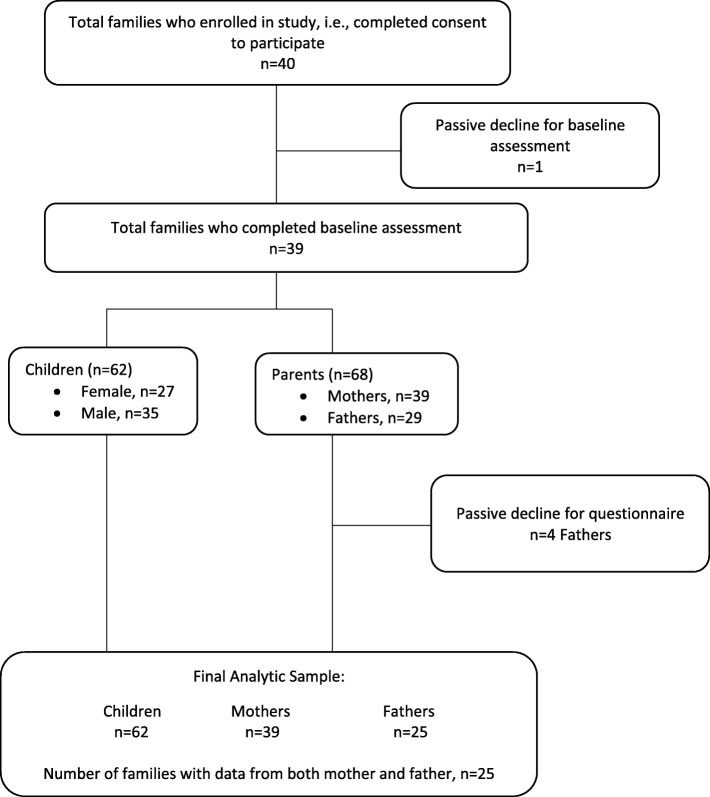
Participant involvement beginning from enrollment to final analytic sample for the Guelph Family Health Study Phase 2 pilot

This study used baseline data collected between February 24, 2016 to December 15, 2016 among parents participating in the GFHS phase 2 pilot study. The GFHS is a family-based cohort study designed to identify early life risk factors for obesity and chronic disease and to test family-based approaches for health promotion. Recruitment efforts included flyers and social media at local organizations who serve families with young children in the Guelph and Wellington County, Ontario, Canada. Families were eligible to participate in the GFHS if they had at least one child aged 1.5–5 years at the time of recruitment, lived in or near Guelph, Ontario, Canada, had a parent who could respond to questionnaires in English, and the children were without severe health conditions that would prohibit participation in study activities. Parents provided informed consent. A total of 39 families (62 children and 68 parents) participated in the GFHS phase 2 pilot. Four fathers did not complete child questionnaires and were therefore not included in this study. The final analytic sample included 39 families, with 25 of those families providing data from both mother and father, resulting in a total of 64 parents (39 mothers and 25 fathers), and 62 children. Approximately 92% of families were two-parent households, and 8% of families were single-parent households. The study was approved by the University of Guelph Research Ethics Board (REB14AP008) (Fig. 1).

### Measures

#### Media parenting practices

To assess media parenting practices, both mothers and fathers separately completed an on-line questionnaire that included purpose-designed items that were informed by previously designed measures to assess media parenting practices [15, 16]. For parents with multiple children, one questionnaire was completed by both mother and father for each child enrolled in the study. The following six parenting practices were assessed: Parental modeling (2 items): “when I am with my child I use a screen based device” and “I try to limit how much I use a screen based device when I am with my child”; Mealtime screen use (2 items): “our family often watches a screen during meals” and “family members are allowed to use screen based devices during meals”; Use of screens in bedroom (3 items): “my child falls asleep while using a screen based device”, “a screen based device is usually playing in the room when my child falls asleep”, and “my child has access to a mobile screen based device in bed”; Use of screens to control behaviour (2 items): “I offer screen time to my child as a reward for good behaviour” and “I take away screen time from my child as a punishment for bad behaviour”; Parental monitoring of screen time (2 items): “I keep track of my child’s screen time during the week” and “I keep track of my child’s screen time during the weekend”; Limit setting (3 items): “I limit my child’s screen time during the week”, “I limit my child’s screen time during the weekend” and “I encourage my child to do activities other than screen time”. Response options for each media parenting practice item included a 4-point Likert scale ranging from “strongly disagree” to “strongly agree.” Responses were coded numerically from 1 to 4 and then totalled to create a score for each media parenting practice, with the exception of limit setting and monitoring, where score for parenting practice was totalled separately for weekday and weekend day. The parenting practice of using screens in the child’s bedroom was endorsed by only one out of 64 parents in the GFHS, phase 2 pilot. Given this lack of variation, the parenting practice of using screens in the bedroom was removed from analysis. Separate Cronbach’s α for mothers and fathers were run to measure internal consistency among questions asked within each of the six parenting practices (Table 1). For parental modeling, there was poor internal consistency (Cronbach’s α for Mothers = 0.12, Fathers = 0.36), resulting in the modelling item “I try to limit how much I use a screen based device when I am with my child” being excluded, and “when I am with my child I use a screen based device” to be examined independently. The remaining four parenting practices showed good internal consistency for both mothers and fathers ranging between 0.72 and 0.95 (Table 1). In addition to measuring these parenting practices, we also assessed parent overall screen time using the same questions used to assess child screen time (described below).

**Table 1:** Mean scores of mothers’ and fathers’ media parenting practices and their Cronbach’s α values

Parenting Practice	Mother Mean (SD)	Father Mean (SD)	Cronbach’s α Mother	Cronbach’s α Father
Screen Time Modeling	2.68 (0.59)	2.55 (0.78)	n/a	n/a
Mealtime Screen use	1.45 (0.58)	1.58 (0.61)	0.72	0.76
Screens to Control Behaviour	1.90 (0.80)	2.00 (0.97)	0.79	0.95
Monitoring Screen Time	2.94 (0.93)	2.95 (0.91)	0.95	0.95
Limiting Screen Time	3.44 (0.56)	3.48 (0.51)	0.85	0.81

#### Child screen time

Parent one, defined as the first parent to enroll in the study, reported on children’s total recreational screen time on an average weekday and weekend day. In the questionnaire, screen time was defined for parents as “any time that is spent on screens such as television, cell phones, iPads or tablets, and videogames.” The specific questions were “not including screen time for school/homework, how many hours does your child spend on screens on an average weekday?” and “not including screen time for school/homework, how many hours does your child spend on screens on an average weekend day?” Response options were none, less than 1 h per day, 2–3 h per day, 4–6 h per day, and 7 or more hours per day. Response options were coded as 0, 1, 2.5, 5, and 7, respectively. Weekday and weekend day screen times were examined separately as research suggests that level of children’s screen time and associations between media parenting practices and children’s screen time may differ between weekdays and weekend days [17].

#### Covariates

Parent one provided the information for household income, number of children in the family, and sex of the child on the baseline questionnaire. Child age was calculated using the child’s birthdate and the date of the child’s baseline assessment.

#### Weight, height, and BMI

Parent and child weight, height and BMI were collected at the University of Guelph in the Body Composition Lab during the baseline health assessment visit. Height was measured using a wall-mounted stadiometer with barefoot or socks and no hair accessories. Two height measurements were taken and if there was a difference greater than 0.5 cm, a third measurement was taken. The final height measurement was a calculated using the average of the two closest measurements. Weight was taken using the BOD POD™ digital scale and were barefoot or in socks. BMI (kg/m^2^) for adults was calculated using the weight measurement in kilograms divided by the average height measurement in meters squared. For children, the BMI z-score was calculated using WHO Anthro [18] and WHO AnthroPlus [19].

#### Statistical analysis

SAS University Edition, version 3.6 [20] was used to perform all analyses. Linear regression using generalized estimating equations was used to determine whether associations exist between media parenting practices (parental modeling, mealtime screen use, use of screens in bedroom, use of screens to control behaviour, parental monitoring of screen time, and limit setting) and children’s screen time (weekend and weekday) while adjusting for correlations among study participants. Analyses were stratified by parent gender and by weekday/weekend day of child screen time. All models included household income, number of children in the family, child sex, and child age. Significance was determined as *p* < 0.05.

## Results

### Sample

Participant demographic information is presented in Table 2. Children had a mean age of 3.65 standard deviation (SD 1.36) years and 57% of the children were male. Parents had a mean age of 37.56 (5.55) years, 61% were female, and 54% of families had a yearly household income of over $100,000. Approximately 87% of children, and 91% of parents identified as Caucasian. Participant children had mean BMI z score of 0.7 (1.04), mothers had a mean BMI of 28 kg/m2 (7.67), and fathers had a mean BMI of 27 kg/m2 (4.50). For children, mean weekday and weekend day screen time measured in hours was 1.24 (0.75) and 1.88 (1.27), respectively. Parents had a mean screen time of 2.03 (1.31) hours during the weekday and 2.34 (1.19) hours during the weekend.

**Table 2:** Characteristics of parents and their preschool-aged children in the Guelph Family Health Study Pilot 2

Variable	Children	Families	Mother	Fathers
	*N* = 62	*N* = 39	*N* = 39	*N* = 25
Total number of children in family, *n* (%)				
1		7 (17.94)		
2		21 (53.85)		
3 or more		11 (28.21)		
Sex, *n* (%)				
Male	35 (56.45)			25 (39.06)
Female	27 (43.55)		39 (60.94)	
Age, y, Mean (SD)	3.65 (1.36)		36.80 (4.75)	38.76 (6.53)
Ethnicity, *n* (%)				
Caucasian	54 (87.1)		35 (89.74)	23 (92.00)
Other	8 (12.9)		4 (10.26)	2 (8.00)
Screen Time, hours				
Weekday Mean (SD)	1.24 (0.75)		2.29 (1.26)	1.74 (0.83)
Weekend Mean (SD)	1.88 (1.27)		2.24 (1.09)	2.20 (1.42)
Weight status, BMI^a^, Mean (SD)	0.66 (1.04)		27.94 (7.67)	27.41 (4.50)
Household Income, *n* (%) of families				
<$40,000		3 (7.69)		
$40,000–$69,999		5 (12.82)		
$70,000–$99,999		9 (23.08)		
$1000 000–$149,999		12 (30.77)		
>$150,000		9 (23.08)		
Did not Answer		1 (2.56)		

^a^BMI (*N* = 60) was used for adult weight status, four women were omitted d/t pregnancy; BMI z score (*N* = 60) was used for child weight status to account for age and sex, two children omitted as unable to obtain height measurement

### Associations between maternal and paternal media parenting practices and children’s total screen time

#### Mother media parenting practices

When examining children’s weekday screen time, mothers’ own use of screens in front of their children (modeling) ($\widehat{\upbeta}$ = 0.42; 95% CI, 0.09 to 0.76; *p* = 0.013), mealtime screen use ($\widehat{\upbeta}$ = 0.21; 95% CI, 0.09 to 0.04; *p* = 0.017), and use of screens to control behaviour ($\widehat{\upbeta}$ = 0.15; 95% CI, 0.01 to 0.29; *p* = 0.038) were positively associated with children’s weekday screen time. Mothers’ practices of monitoring screen time ($\widehat{\upbeta}$ = − 0.34; 95% CI, − 0.53 to − 0.15; *p* < 0.001), and limiting screen time ($\widehat{\upbeta}$ = − 0.31; 95% CI, − 0.47 to − 0.15; *p* < 0.001) were inversely associated with young children’s weekday screen time (Table 3). Results for children’s weekend screen time yielded similar results for mothers’ media parenting practices with two exceptions. Mothers’ mealtime screen use and modeling screen time were not significantly associated with children’s weekend screen time (Table 4). No association was found between mothers’ weekday or weekend overall screen time and children’s weekday or weekend screen time (Tables 3 and 4).

**Table 3:** Results of linear regression modeling using GEE investigating effects of media parenting practices on children’s weekday screen-time

Parenting Practice	Parent	Child Screen Time Weekday	
		Adjusted estimate^a^ (95% CI)	*P*-value
Screen Time Modeling	Mother	$\widehat{\boldsymbol{\upbeta}}$ **= 0.42 (0.09, 0.76)**	**0.01**
	Father	$\widehat{\upbeta}$ = −0.05 (− 0.43, 0.33)	0.81
Mealtime Screen Use	Mother	$\widehat{\boldsymbol{\upbeta}}$ **= 0.21 (0.09, 0.04)**	**0.02**
	Father	$\widehat{\boldsymbol{\upbeta}}$ **= 0.21 (0.03, 0.40)**	**0.02**
Screens to Control Behaviour	Mother	$\widehat{\boldsymbol{\upbeta}}$ **= 0.15 (0.01, 0.29)**	**0.04**
	Father	$\widehat{\upbeta}$ = 0.09 (−0.04, 0.23)	0.17
Monitoring Screen Time	Mother	$\widehat{\boldsymbol{\upbeta}}$ **= −0.34 (−0.53, − 0.15)**	**< 0.01**
	Father	$\widehat{\boldsymbol{\upbeta}}$ **= −0.40 (− 0.62, − 0.19)**	**< 0.01**
Limiting Screen Time	Mother	$\widehat{\boldsymbol{\upbeta}}$ **= −0.31 (− 0.47, − 0.15)**	**< 0.01**
	Father	$\widehat{\boldsymbol{\upbeta}}$ **= −0.44 (− 0.61, − 0.27)**	**< 0.01**
Weekday Screen Time	Mother	$\widehat{\upbeta}$ = 0.09 (− 0.08, 0.26)	0.28
	Father	$\widehat{\upbeta}$ = 0.08 (− 0.25, 0.41)	0.64

^a^Adjusted for total number of children in the family, family income, child sex, and child age

Bolded values indicate significance

**Table 4:** Results of linear regression modeling using GEE investigating effects of media parenting practices and children’s weekend day screen-time

Parenting Practice	Parent	Child Screen Time Weekend	
		Adjusted estimate^a^ (95% CI)	*P*-value
Screen Time Modeling	Mother	$\widehat{\upbeta}$ = 0.47 (−0.06, 0.10)	0.08
	Father	$\widehat{\upbeta}$ = −0.03 (− 0.77, 0.72)	0.94
Mealtime Screen use	Mother	$\widehat{\upbeta}$ = 0.24 (−0.01, 0.50)	0.06
	Father	$\widehat{\upbeta}$ = 0.07 (−0.25, 0.38)	0.70
Screens to Control Behaviour	Mother	$\widehat{\boldsymbol{\upbeta}}$ **= 0.37 (0.12, 0.61)**	**< 0.01**
	Father	$\widehat{\boldsymbol{\upbeta}}$ **= 0.31 (0.04, 0.57)**	**0.02**
Monitoring Screen Time	Mother	$\widehat{\boldsymbol{\upbeta}}$ **= −0.44 (−0.86, −0.01)**	**0.045**
	Father	$\widehat{\boldsymbol{\upbeta}}$ **= −0.48 (−0.89, − 0.08)**	**0.02**
Limiting Screen Time	Mother	$\widehat{\boldsymbol{\upbeta}}$ **= −0.41 (−0.72, − 0.10)**	**0.01**
	Father	$\widehat{\boldsymbol{\upbeta}}$ **= −0.58 (−1.02, − 0.13)**	**0.01**
Weekend Screen Time	Mother	$\widehat{\upbeta}$ = −0.12 (− 0.36, 0.12)	0.32
	Father	$\widehat{\upbeta}$ = 0.29 (−0.01, 0.59)	0.06

^a^Adjusted for total number of children in the family, family income, child sex, and child age

Bolded values indicate significance

#### Father media parenting practices

Fathers’ practice of using screens during mealtime ($\widehat{\upbeta}$ = 0.21; 95% CI, 0.03 to 0.40; *p* = 0.023) was positively associated with children’s weekday screen time. Fathers’ practice of monitoring screen time ($\widehat{\upbeta}$ = − 0.40; 95% CI, − 0.62 to − 0.19; *p* < 0.001), and limit setting ($\widehat{\upbeta}$ = − 0.44; 95% CI, − 0.61 to − 0.27; *p* < 0.001) were inversely associated with children’s weekday screen time. Fathers’ screen time modeling, using screens to control behaviour, and overall screen time were not significantly associated with children’s weekday screen time (Table 3). Results for children’s weekend screen time yielded similar results for fathers’ media parenting practices with two exceptions. Fathers’ practice of mealtime screen use was not significantly associated with children’s total weekend screen time, but fathers’ use of screens to control behaviour ($\widehat{\upbeta}$ = 0.31; 95% CI, 0.04 to 0.57; *p* = 0.024) was positively associated with children’s weekend screen time (Table 4).

## Discussion

This study examined the associations between media parenting practices and young children’s screen time among a sample of Canadian families with children aged 1.5–5 years. Overall, this sample had a lower rate of overweight and obesity [21], and a higher median income compared to the Canadian Population [22]. The results show that the media parenting practices of both mothers and fathers influence the amount of time children spend in front of screens. Regression coefficient estimates for each significant association range from 0.15 to 0.58, translating to between 9 and 35 min of children’s screen time per day, per unit increase in the respective parenting practice, which suggests these parenting practices have a meaningful impact on children’s screen time. For weekday mealtime screen use, monitoring screen time and limit setting, and weekend using screens to control behaviour and monitoring screen time the regression coefficient estimates remain relatively consistent, suggesting that both mothers and fathers may have a similar influence on children’s screen time.

The parenting practice of mealtime screen use for both mothers and fathers resulted in children spending more time during the weekdays in front of a screen-based device. These results support previous research from the Canadian TARGet Kids! study, which found that one of the factors significantly associated with increased total daily screen time was television viewing during meals [23]. Taken together, these findings suggest that interventions focused on reducing mealtime screen use has the potential to reduce children’s overall screen time.

Both mother’s and fathers’ use of screens to control behaviour was positively associated with children’s screen time. These results mirror what has been found in research examining parental influences on children’s physical activity and dietary intake. Research has shown that when parents use food to control behaviour, children have a greater dietary intake of those same foods [24]. This is also true for physical activity. A study by Vaughn et al. [16] found that the use of physical activity as a reward for good behaviour was positively associated with physical activity. Taken together, these results suggest that using physical activity, food, or screen time to control children’s behaviours may lead to higher levels of those same behaviours. Alternatively, it may be that when parents are trying to control their children’s behaviour, they choose a reward or punishment that they know their child values, i.e., they only select screen time as a reward when their child really values screen time. Children who really value screen time may watch more screen time, in general. Thus, this association between parental control and screen time may be partly due to reverse causation. Longitudinal research is needed to help determine temporal order of parenting practices and children’s screen time.

The association between father’s use of screens to control child behaviour and child screen time was significant only for children’s weekend screen time. This may be due to the greater availability and involvement of fathers with their children on weekends as compared to weekdays. This increase in weekend involvement is described in a study by Yeung et al. [25], who found that fathers of intact families have 6.5 h of involvement time with their children on a weekend day, compared to 2.5 h on a weekday. This study also determined that the amount of time fathers are engaged or accessible to children between 3 and 5 years of age is 58% that of mothers on weekdays, and 86% that of mothers on weekends [25], potentially further explaining why fathers’ use of screens to control behaviour is significant on weekends and not weekdays. However, more studies that include fathers are needed to validate this result as there is only one other study that has examined this parenting practice. Among their sample of parents (93% mothers) Vaughn and colleagues found that removing television time as a punishment and using television to control a child’s behaviour was positively associated with children’s overall TV time [16].

This study was the first to investigate the association between the media parenting practice of monitoring screen time and children’s total screen time. For both mothers and fathers, monitoring the amount of screen time was inversely associated with children’s weekday and weekend total screen time. These results demonstrate that parental monitoring of screen time could potentially change screen time viewing behaviour among young children and may be an important target for interventions.

Our findings support previous research, which has shown that the media parenting practice of limit setting is successful in reducing children’s total screen time [26–28]. The majority of existing studies examined mother’s parenting practices only. One study that examined the parenting practices of mothers and fathers among 70 Australian families of school aged children found that the media parenting practice of limit setting for fathers, but not mothers, was inversely associated with children’s screen time [28]. This study builds on previous research that examined fathers’ influence and found that limit setting by both mothers and fathers is inversely associated with screen time. Thus, intervention approaches aiming to reduce children’s screen time should address limit setting practices among both mothers and fathers.

No association was found between the overall weekday and weekend day screen time of mothers or fathers with children’s weekday and weekend day screen time. This finding may be due to the young age of children in this study. A study by Carson and Janssen [29] found that parental screen time was associated with the screen time of children between 4 and 5 years of age, but not among children from birth to 3 years. It is possible that parents of young children have more opportunity to engage in screen time away from their children, i.e., during nap time or an early bed time. As children age and they nap less and go to bed later, this additional awake time may result in increased screen time for both parents and children. Modelling of screen time, i.e., using screens in front of their children, was positively associated with children’s weekday and weekend screen time among mothers, but not among fathers. These results supported those of Matarma et al. [12] who found that television time of mothers, but not fathers, was positively associated with their children’s television time. This could potentially be because although there has been a shift in the diversity of family structures, mothers remain the primary caregiver in the majority of families [30], and are therefore spending more time with their children. Thus, it is reasonable to believe that mothers would exert more influence on their children through modeling behaviours when compared to fathers. As mentioned previously, additional research that explores fathers’ influence on children’s screen time is needed to confirm our findings.

Only one out of 64 parents endorsed screen use in the bedroom, and therefore we were unable to move forward with analysis of this variable. A potential explanation for the low practice of using screens in the bedroom could be due to the relatively high socioeconomic status (SES) of participants in this study, as this parenting practice may not be as common among families with higher SES [31]. It also may not be as common for Canadian children to have a screen based device in their bedroom, when compared to in the United States (U.S.), where over half of American children have a television in their bedroom [32]. This may point to a potential cultural difference related to the parenting practice of using screens in the bedroom between the U.S. and Canada.

Strengths of our study included the inclusion of mobile media devices such as tablets and smartphones in our assessment of children’s screen time. This allowed us to investigate how media parenting practices influence children’s screen based sedentary behaviours in a way that represents the current media environment. Second, this study included the practice of monitoring screen time and use of screens to control behaviour, parenting practices that are limited in the current research. This allowed for better understanding of the role a range of parenting practices have on total screen time in young children. Lastly, the exploration of the impact of fathers’ parenting practices helps address a key gap in our understanding of influences on children’s screen time behaviours.

This study had some limitations that should be considered when interpreting our results. First, both parenting practices and children’s screen time were based on parent report. This may result in social-desirability bias or errors in estimating children’s daily average screen time. Further, self-reported measures of screen time have variable levels of validity for both children and adults when compared to objective measures. A second limitation is the relatively high socioeconomic status and that most of the participants identify as Caucasian. Therefore, results may not be generalizable to ethnically diverse families or families with lower SES. Lastly, there was no data collected on current work status of parents. This information would help to determine time spent with children, which is important when considering parenting practices such as modeling and using screens to control behaviour. Future research should examine these associations using objective measures of screen time within more ethnically and racially diverse populations.

The results of this study provide a more complete understanding of the influence media parenting practices have on young children’s screen time. It is important that children’s screen time recommendations are met, as exceeding screen time recommendations can negatively affect young children’s cognitive and emotional development, attention span, and future academic performance [33, 34] and increase the risk of childhood obesity [6, 7]. Results from this study can be used to guide parents in meeting screen time recommendations that promote positive health and development among their young children.

## Conclusion

Overall, media parenting practices used by both mothers and fathers was associated with young children’s total screen time. Given that parents play a key role in the development of their young children’s screen time behaviours, and that research shows exceeding screen time recommendations may have negative physical, developmental, and psychosocial affects on young children [33, 34], fully understanding the influence of both mother and father media parenting practices has on children’s screen time in the current media environment is needed. This knowledge will guide parents toward media parenting practices that work to achieve the current screen time recommendations. This study furthers that understanding by highlighting the importance of targeting both mothers’ and fathers’ media parenting practices. Further study is needed that includes mobile media devices in the assessment of children’s screen time, examines the relatively understudied media parenting practices of monitoring screen time and using screens to control behaviour, and that includes a more ethnically diverse population. Research that further explores the role of fathers’ media parenting practices on children’s screen time is also needed.

## Abbreviations

BMI: Body Mass Index
GEE: Generalized Estimating Equations
GFHS: Guelph Family Health Study
SES: Socioeconomic Status
TV: Television
U.S.: United States
WHO: World Health Organization
